# Community-based survey during rabies outbreaks in Rangjung town, Trashigang, eastern Bhutan, 2016

**DOI:** 10.1186/s12879-017-2393-x

**Published:** 2017-04-17

**Authors:** Tenzin Tenzin, Jamyang Namgyal, Sangay Letho

**Affiliations:** 1 Disease Prevention and Control Unit, National Centre for Animal Health, Department of Livestock, Thimphu, Bhutan; 2 District Veterinary Hospital, Department of Livestock, Trashigang, Bhutan; 3 Regional Livestock Development Centre, Department of Livestock, Khangma, Trashigang, Bhutan

**Keywords:** Community survey, Rabies outbreak, Rapid response team, Trashigang, East Bhutan

## Abstract

**Background:**

Rabies is a highly fatal disease transmitted through the bite of a rabid animal. Human deaths can be prevented by prompt administering of rabies vaccine and rabies immunoglobulin following the exposure. An assessment of community knowledge, awareness and practices on rabies is important during outbreak to understand their preparedness and target educational messages and response activities by the rapid response team.

**Methods:**

A rabies outbreak has occurred in Rangjung town, eastern Bhutan on 4 October 2016. A rapid response team was activated to investigate outbreak and to establish a control program. A community-based questionnaire survey was conducted from 20 to 21 October 2016 to assess the community knowledge of rabies to guide outbreak preparedness and also target educational messages and response activities by the RRT.

**Results:**

A total of 67 respondents were interviewed, of which 61% were female and 39% male. All the respondents have heard of rabies (100%), have knowledge on source of rabies (dog) and its mode of transmission in animals and humans. Most (61%) respondents were aware and also indicated that they would wash the animal bite wound with soap and water and seek medical care on the same day of exposure (100%). Majority (94%) of the respondents have indicated that they would report to the government agencies if they see any suspected rabid dogs in the community and suggested various control measures for dog population management and rabies in Rangjung including neutering procedure and mass dog vaccination. Although only few (10%) of the respondents households owned dogs and cats, but 50% of them have indicated that their dogs were allowed to roam outside the home premises posing risk of contracting rabies through rabid dog bites.

**Conclusions:**

Although this study indicates a high level of knowledge and awareness on rabies among the community, there exists some knowledge gaps about rabies and therefore, an awareness education should be focused on the source of rabies and rabies virus transmission route to reduce public concern on nonexposure events thereby reducing the cost on unnecessary postexposure treatment.

**Electronic supplementary material:**

The online version of this article (doi:10.1186/s12879-017-2393-x) contains supplementary material, which is available to authorized users.

## Background

Rabies is a highly fatal disease mainly transmitted through the bite of a rabid animal. Canine mediated rabies is endemic in many developing countries causing an estimated 59,000 human death annually [1]. Sustained mass dog vaccination covering >70% of the dog population is the only feasible methods to eliminate the transmission cycle of rabies virus in dogs [2, 3]*.*

**Fig. 1:**
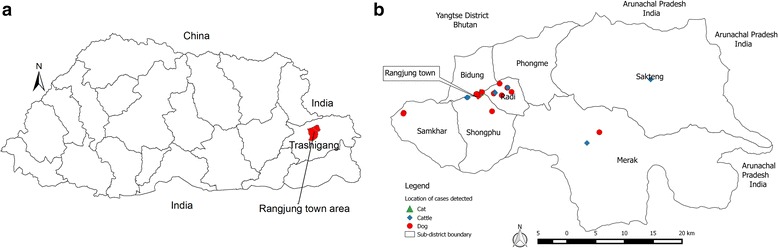
Bhutan map showing the location of Rangjung town under Trashigang District in eastern Bhutan (Panel **a**) and the approximate location of rabies cases detected in domestic animals in Rangjung town and the surrounding villages (4 Oct 2016 to 8 Jan 2017) (Panel **b**). The first cases detected in a stray dog and yaks at Merak and Sakteng sub-districts during July–August 2016 which may be the source of current outbreak in Rangjung town and its surrounding villages is also shown on the map

In Bhutan, rabies is endemic in southern parts of the country that borders with India and results in sporadic deaths of domestic animals and humans following rabid dog bites [4, 5]*.* However, sporadic incursions or spread from the south border towns into the interior rabies free areas have occurred in the recent past, indicating possible re-emergence of rabies in the country [6, 7]*.* On 4 October 2016, a stray dog suspected of rabies became aggressive and had bitten 3 people in Rangjung town, Trashigang district in eastern Bhutan (Fig. 1). In the subsequent days (until 8 January 2017), 10 other stray dogs in Rangjung showed aggressive behavior and were either died of the disease or captured and euthanized. A multisectoral Rapid Response Team (RRT) from animal and public health agencies have visited the outbreak area to investigate and to establish a control program. A case of rabies was suspected if an animal demonstrate change in behavior and become very excitable, aggression, excessive salivation, biting unusual objects like sticks and stones, aimless movement, and paralysis in case of dog and cat, and excessive salivation, behavioral changes, vocalization (bellowing), aggression, hyperesthesia, paralysis, coma and death in case of cattle; a case of rabies was confirmed if the brain tissue samples tested positive to rabies virus by rapid antigen detection test (BioNote) and fluorescent antibody test (FAT) [8]*.* As of 8 January 2017, 18 dog, 1 cat and 6 cattle were confirmed to have been infected with rabies virus in Rangjung town and in the surrounding villages*.* The movement of infected dogs and subsequent bites to other susceptible animals have resulted in the spread of outbreak. The RRT had implemented the control measures including: tracing, capture and euthanasia of suspected rabies dogs, collection of brain tissue samples and conduct of laboratory test; zoo-sanitary measures (safe disposal of carcasses through burial); an emergency vaccination of susceptible dogs and cats to create immune buffer; active surveillance; and awareness education to the general public and the students. A total of 5 people in Rangjung town and 7 in the surrounding villages were bitten by confirmed rabid dogs and cat. All the bite victims have received post exposure treatment including wound washing with soap and water/antiseptic dressing; infiltration of human rabies immunoglobulin (HRIGs) around the bite site and received complete course of WHO recommended intradermal rabies vaccine (purified chick embryo cell vaccine (PCECV) - Rabipur) on day 0, 3, 7 and 28 as per the rabies postexposure treatment guideline.

In order to understand the community’s knowledge, awareness and reaction to rabies outbreak, a quick questionnaire survey was conducted among the residents of Rangjung town in October 2016. An assessment of community knowledge of rabies can help to understand their preparedness and also target educational messages and response activities by the rapid response team.

## Methods

### Study area

Rangjung is a small satellite town situated 17 km north of the district headquarter Trashigang (Fig. 1). The core town area (0.134 km^2^) is administratively under the control of Rangjung municipality. There are shops (*n* = 41) and residential houses within the core town area and the schools, religious institution, health centres, corporate offices and also residential houses (*n* = 100) outside the core town boundary in Rangjung. Approximately 438 people (including children) live within the core town area and another 1576 people (including children) live outside the core town boundary, with vast majority being students and monks. Rangjung is also a central area in the north of Trashigang where people from the surrounding villages of the six sub-district visit daily on their way to and fro from district headquarter Trashigang.

### Data collection

A questionnaire survey was conducted among the residents of Rangjung town from 20 to 21 October 2016 to assess the community knowledge on rabies to guide outbreak preparedness and also target educational messages and response activities by the RRT. The questionnaire included closed questions related to respondent information, knowledge and awareness on rabies, practices and attitude towards dog population management and rabies control; dog bite details and wound management, and dog ownership (Additional file 1). Since this survey was conducted as part of an emergency response during the peak of an outbreak by RRT, no ethical approval was necessary. Moreover, the surveys were conducted anonymous and were not linked to a name or address of the respondents. The participants for the interview included businessmen, government or private employee and farmers residing in Rangjung town (one per selected household). The selected person was informed about the purpose of the study and that participation was voluntary and data collected were confidential. The participants were recruited after the conduct of public meeting and all the approached person (*n* = 67) have agreed for the interview.

### Data analysis

The data were entered into a database developed in EpiInfo™ version 7.1.2.0 (Centers for Disease Control and Prevention (CDC), Atlanta, GA, USA) and analyzed using Stata version 13.1 (Stata, 2013). Descriptive statistics were calculated for each variable of interest to compare responses to questions related to the knowledge, attitudes and practices of rabies.

## Results

A total of 67 respondents from 67 household were contacted and interviewed, of which 61% were female and 39% male. Most respondents had lived in Rangjung town for more than 5–10 years (64%), mostly running business (45%). All the respondents have heard of rabies (100%), known the source of rabies (dog) and its mode of transmission in animals and humans. Most have got the information through friends/neighbours and through awareness education conducted by the veterinary and public health officials (Additional file 1). Most (61%) respondents were aware and also reported that they would wash the animal bite wound with soap and water and seek medical care on the same day of exposure (100%) (Additional file 1). However, among this study respondents, only 11 person (16%) were bitten by dogs during their life time (at the time of survey; until 21 October 2016) and most were bitten by stray dogs (64%) (Additional file 1). Of these, 3 person were bitten by confirmed rabid dogs in Rangjung during recent outbreak and all have received complete PEP course including human rabies immunoglobulin.

Majority (94%) of the respondents have indicated that they would report to the government agencies (the veterinary, municipal, public health, community representative) if they see any suspected rabies animals in the community, but most (64%) were not willing to kill the rabid dogs. They would want the government agencies to control rabies in Rangjung through various means including killing/removal, catching and impounding of stray dogs in one place, catching and translocating dogs, and through emergency vaccination program (Additional file 1). The respondents (58%) have also indicated the problem of stray dogs in Rangjung town and suggested neutering procedure (65%) to control dog population and rabies in future. Only 10% of the respondents owned dogs and cats (7 household owned dogs and 6 HH owned cats), but 50% of them indicated that their dogs were allowed to roam outside the home premises day and night (Additional file 1).

## Discussion

This study was conducted during the time of rabies outbreak in Rangjung town to understand and assess the community knowledge, attitudes and perception of rabies to help to prepare and also target educational messages and response activities by the rapid response team. The study indicated high level of knowledge and awareness on rabies amongst the respondents in Rangjung town which may be attributed to repeated visits by the RRT for emergency mass dog vaccination campaign, contact-tracing of the rabid suspected dogs and dog bite victims in humans, continuous consultation and education of the people on rabies by the RRT. Moreover, Rangjung town reported an outbreak of rabies during 2006 where more than 400 people were administered rabies post-exposure prophylaxis (PEP) as a result of contact with rabies suspected animals and also consumption of milk and dairy products derived from rabies suspected and rabid confirmed cows [6]*.* The past outbreak incident and the educational messages given by the RRT may likely had a positive effect on the extent of knowledge retained by people in Rangjung [6]*.* However, there exist some knowledge gaps particularly related to the source and mode of transmission of rabies. For instance, a large number of respondents have not given correct answers to the questions and stated that contact with urine and feces, insect bites, touching animals and consumption of animal products would result in the transmission of rabies. Such misconceptions have resulted in unnecessary PEP administration and increased the public cost since the people would visit the hospital and ask for PEP even if the risk is low or negligible [9, 10]*.* During the current outbreak in Rangjung, 92 people have requested and been administered anti-rabies vaccine because they have touched and fed a dog that later disappeared from the area. Therefore, it is important to educate the community on specific source of vectors such as what animals can get rabies and also the routes of exposure of rabies virus so that there is no misconceptions about rabies transmission from noninfectious routes, thereby reducing the public concern and also reduce the cost of PEP. The community were aware on the importance of washing the animal bite wound with soap and water and seeking medical care on the same day of exposure. This findings is in agreement with the knowledge and attitude of other community in south Bhutan [11]*.* The first aid measures (e.g. washing bite wound with soap and water) and treatment seeking-behaviors is important for rabies prevention in humans since majority of the people in the developing countries die of rabies due to failure to seek medical care in time [12]*.* The hospital record of Rangjung health centre also indicated visits of people for rabies PEP even for non-bite exposures. During the current outbreak in Rangjung and surrounding areas, all persons (*n* = 12) bitten by confirmed rabid dogs and cat have visited the health centres and received complete PEP course including human rabies immunoglobulin. The health seeking behavior of people in Bhutan for rabies PEP is high when compared to other developing countries [4, 9–12]*.* This may be due to availability of free health care services in the country including rabies PEP. However, it is to be noted that there were instance of human deaths due to rabies in the south Bhutan region (17 people died of rabies between 2006 and 2016), especially in children, who did not visit the health centre for PEP [4]*.* During the current outbreak in Rangjung, the RRT have ensured to trace the dog bite victims and referred to the hospital for treatment. In addition, awareness education on rabies and its prevention and control aspects were provided by the RRT to the general public and to the teachers and students in the outbreak areas.

Our study also demonstrated the attitude of the respondents where majority (94%) have indicated that they would report to the government agencies (the veterinary, municipal, public health, community representative) if they see any suspected rabies animals in the community. The community suggested the government agencies to control rabies in Rangjung through various means including killing, catching and impounding of stray dogs in one place, catching and translocating dogs, and through emergency vaccination program. Surprisingly, some of the respondents would not want to suggest the control measures because they thought that the RRT would implement their suggestions thereby resulting in sinful act since killing is considered sin as per the Buddhist beliefs [6]*.* Translocation and impounding of dogs although suggested by the community is not recommended as it will threatens the success of control programs by spreading rabies. The importance of avoiding or stopping the translocation of dogs was emphasized to the community since one of the reasons for high density of free-roaming/stray dogs in the urban areas was believed to be due to translocation of dogs from rural villages. Therefore, public education should emphasize the risks of translocation of dogs to rabies free areas. However, as control measures, the RRT have carried out an emergency mass dog vaccination [1600 dogs and cats] in the periphery of the outbreak zone and in high risk villages to create immune buffer, and also captured and euthanized the rabies suspected and confirmed dogs to break the transmission cycle. Similarly, since stray dog pose problem and threat to the residents in Rangjung, the community suggested neutering (65%) procedure to control dog population and rabies in future. Bhutan have been implementing catch-neuter-vaccinate-release (CNVR) program to control dog population and rabies [13]. It is important that dog population management campaign including mass dog vaccination against rabies be conducted annually in Rangjung town and the bordering areas in eastern Bhutan to create immune buffer against rabies incursion into the country by strictly following the National Rabies Prevention and Control Plan [14]*.*

Among the respondents, only 10% of the household owned pet dogs and cats, but 50% of them indicated that their dogs were allowed to roam outside the home premises day and night indicating lack of ownership. It is important to note that lack of pet ownership is a serious concern in the country that contributes to free-roaming/stray dog population as well as rabies infection [13, 15]*.* During the recent outbreak in Rangjung town and in the surrounding villages, of the 18 rabies cases in dogs, 2 were confirmed in pet dogs and 1 in pet cat, while 6 pet dogs and 1 cat that were free-roaming were bitten by rabid dogs indicating possible risk of human exposure (household members especially children) and also act as source of infection to other dogs and domestic animals. Thus, it is important to regulate pet ownership in the country through awareness education and enforcement of rules and regulation.

## Conclusions

The findings of this study provide useful information to the veterinary and public health officials to focus and reinforce on specific educational awareness on rabies, particularly the main source of rabies and virus transmission route that will help to reduce public concern about non-exposure events thereby reducing the unnecessary PEP and the cost of treatment. Further, the importance of treatment seeking aspects should also be focused to avoid human deaths from rabies as Bhutan gears towards elimination of dog mediated human rabies by 2020. The importance of public cooperation and support during the time of mass dog vaccination campaign (CNVR) and dog ownership should be emphasized during educational awareness campaign. Thus, the information from this study will be helpful in the event of a future rabies outbreak in Rangjung and elsewhere in the country.

## Additional file

Additional file 1: Demographic characteristics and Rabies-related responses of the respondents. (DOCX 1921 kb)

## Abbreviations

CNVR: Catch-neuter-vaccinate-release
RRT: Rapid Response Team
