# Supplementary material for: Community-based survey during rabies outbreaks in Rangjung town, Trashigang, eastern Bhutan, 2016
Source: BMC Infect Dis. 2017 Apr 17;17:281. doi: 10.1186/s12879-017-2393-x (PMC5393039; doi:10.1186/s12879-017-2393-x)
Supplement: Additional file 1: — Demographic characteristics and Rabies-related responses of the respondents. (DOCX 1921 kb) [file 12879_2017_2393_MOESM1_ESM.docx]

| **Supplementary file 1: Demographic characteristics and Rabies-related responses of the respondents** | | |
| --- | --- | --- |
|  |  |  |
| **Part I: Respondent information and characteristics** |  |  |
| **Variables** | **N** | **Percent** |
| **Gender** |  |  |
| Female | 41 | 61.19 |
| Male | 26 | 38.81 |
| **Age (years)** |  |  |
| 18-30 | 19 | 28.36 |
| 30-40 | 29 | 43.28 |
| >40 | 19 | 28.36 |
| **Occupation** |  |  |
| Businessman | 30 | 44.78 |
| Government employee | 19 | 28.36 |
| Dependent/house wife | 6 | 8.96 |
| Farmers | 12 | 17.91 |
| **Number of years lived in Rangjung town** |  |  |
| upto 5 years | 24 | 35.82 |
| 5-10 years | 10 | 14.93 |
| 10-15 years | 5 | 7.46 |
| 15-20 years | 8 | 11.94 |
| 20-25 years | 9 | 13.43 |
| 25-30 years | 7 | 10.45 |
| above 30 years | 4 | 5.97 |
|  |  |  |
| **Part II: Respondents level of knowledge and awareness about rabies** |  |  |
|  |  |  |
| **Variables** | **N** | **Percent** |
| **Have you heard of rabies** |  |  |
| No | 0 | 0 |
| Yes | 67 | 100 |
| **Source of information on rabies** |  |  |
| Media | 7 | 10.45 |
| Friends/neighbours | 32 | 47.76 |
| Awareness education by veterinary and public health officials | 23 | 34.33 |
| All of the above methods | 5 | 7.46 |
| **When did you hear about rabies** |  |  |
| Heard during recent awareness meeting in Rangjung | 19 | 28.36 |
| Heard before itself | 48 | 71.64 |
| **Have you heard of rabies outbreak in Rangjung town** |  |  |
| No | 0 | 0 |
| Yes | 67 | 100 |
| **Are you concerned about rabies outbreak in Rangjung town** |  |  |
| No | 0 | 0 |
| Slightly | 1 | 1.49 |
| Yes | 66 | 98.51 |
| **What are your main concern** |  |  |
| Human may get rabies | 44 | 65.67 |
| Both human and animal may get rabies | 23 | 34.33 |
| **How fatal and severe is rabies** |  |  |
| Not sure | 2 | 2.99 |
| Severe & fatal disease | 65 | 97.01 |
| **Is rabies treatable/curable once symptom is shown by patients** |  |  |
| No | 35 | 52.24 |
| Not sure | 22 | 32.84 |
| Yes | 10 | 14.93 |
| **Is there any traditional treatment method for dog bite wound and rabies in humans** |  |  |
| No | 18 | 26.86 |
| Not sure | 17 | 25.37 |
| Yes | 32 | 47.76 |
| **Do you believe or have knowledge that the following animals will get rabies?** |  |  |
| **Dog** |  |  |
| No | 0 | 100 |
| Not sure | 0 | 0 |
| Yes | 67 | 100 |
| **Cat** |  |  |
| No | 2 | 2.99 |
| Not sure | 3 | 4.48 |
| Yes | 62 | 92.54 |
| **Livestock (cattle, sheep, goat etc)** |  |  |
| No | 3 | 4.48 |
| Not sure | 5 | 7.46 |
| Yes | 59 | 88.06 |
| **Horse** |  |  |
| No | 4 | 5.97 |
| Not sure | 10 | 14.93 |
| Yes | 53 | 79.1 |
| **Bat** |  |  |
| No | 20 | 29.85 |
| Not sure | 27 | 40.3 |
| Yes | 20 | 29.85 |
| **Wild dog species** |  |  |
| No | 6 | 8.96 |
| Not sure | 13 | 19.4 |
| Yes | 48 | 71.64 |
| **Wild cat species** |  |  |
| No | 6 | 8.96 |
| Not sure | 15 | 22.39 |
| Yes | 46 | 68.66 |
| **Rat/Rodents** |  |  |
| No | 11 | 16.42 |
| Not sure | 20 | 29.85 |
| Yes | 36 | 53.73 |
| **Bird species** |  |  |
| No | 18 | 26.87 |
| Not sure | 16 | 23.88 |
| Yes | 33 | 49.25 |
| **Knowledge and awareness of the respondents on whether humans can contact rabies through exposure by following means to a rabid animals** |  |  |
| **Dog bite** |  |  |
| No | 0 | 0 |
| Not sure | 0 | 0 |
| Yes | 67 | 100 |
| **Cat bite** |  |  |
| No | 0 | 0 |
| Not sure | 1 | 1.49 |
| Yes | 66 | 98.51 |
| **Bite by livestock** |  |  |
| No | 3 | 4.48 |
| Not sure | 5 | 7.46 |
| Yes | 59 | 88.06 |
| **Bite by wild animals** |  |  |
| No | 3 | 4.48 |
| Not sure | 10 | 14.93 |
| Yes | 54 | 80.6 |
| **Scratch by animals** |  |  |
| No | 7 | 10.45 |
| Not sure | 9 | 13.43 |
| Yes | 51 | 76.12 |
| **Touching to animals** |  |  |
| No | 17 | 25.37 |
| Not sure | 14 | 20.9 |
| Yes | 36 | 53.73 |
| **Contact with saliva** |  |  |
| No | 3 | 4.48 |
| Not sure | 3 | 4.48 |
| Yes | 61 | 91.04 |
| **Contact with blood** |  |  |
| No | 9 | 13.43 |
| Not sure | 11 | 16.42 |
| Yes | 47 | 70.15 |
| **Contact with urine and feces** |  |  |
| No | 16 | 23.88 |
| Not sure | 15 | 22.39 |
| Yes | 36 | 53.73 |
| **Licking on skin** |  |  |
| No | 2 | 2.99 |
| Not sure | 13 | 19.4 |
| Yes | 52 | 77.61 |
| **Eating meat** |  |  |
| No | 3 | 4.48 |
| Not sure | 9 | 13.43 |
| Yes | 55 | 82.09 |
| **Consumption of dairy products (butter, cheese, whey)** |  |  |
| No | 0 | 0 |
| Not sure | 9 | 13.43 |
| Yes | 58 | 86.57 |
| **By milking a rabid cow** |  |  |
| No | 5 | 7.46 |
| Not sure | 7 | 10.45 |
| Yes | 55 | 82.09 |
| **Drinking milk** |  |  |
| No | 1 | 1.49 |
| Not sure | 5 | 7.46 |
| Yes | 61 | 91.04 |
| **Insect bite** |  |  |
| No | 15 | 22.39 |
| Not sure | 18 | 26.87 |
| Yes | 34 | 50.75 |
| **Are you aware that the animal bite wound should be washed with soap and water** |  |  |
| No | 6 | 8.96 |
| Yes | 61 | 91.04 |
| **How long should the bite wound be washed with soap and water** |  |  |
| Just spash water to the bite wound for few seconds | 5 | 7.46 |
| Less than 5 minutes | 18 | 26.87 |
| Between 5 to 10 minutes | 17 | 25.37 |
| Between 10 to 15 minutes | 9 | 13.43 |
| More than 15 minutes | 12 | 17.91 |
|  |  |  |
| **Part III: Dog bite in humans and treatment** |  |  |
|  |  |  |
| **Variables** | **N** | **Percent** |
| **Have you been bitten by dog or not** |  |  |
| No | 56 | 83.58 |
| Yes | 11 | 16.42 |
| **Ownerhsip of biting dog** |  |  |
| Pet dog | 4 | 36.36 |
| Stray dog | 7 | 63.64 |
| **Body parts bitten** |  |  |
| Leg/foot | 8 | 72.73 |
| Thigh | 2 | 18.18 |
| Trunk (back of the body) | 1 | 9.09 |
| **Circumtances of bite** |  |  |
| Provoke bite | 4 | 36.36 |
| Unprovoke bite | 7 | 63.64 |
| **Health status of the biting dog** |  |  |
| Rabies suspected | 3 | 27.27 |
| Apparently healthy and normal dog | 8 | 72.73 |
| **Severeity of the bite wound** |  |  |
| Single bite wound | 4 | 36.36 |
| Multiple bite wound | 5 | 45.45 |
| Transdermal scratches | 2 | 18.18 |
| **Number of times bitten by dog in life time (till date)** |  |  |
| 1 time | 8 | 72.73 |
| 3 time | 1 | 9.09 |
| 4 time | 1 | 9.09 |
| 5 time | 1 | 9.09 |
| **Place of bite** |  |  |
| Rangjung town | 6 | 54.55 |
| Other places | 4 | 36.36 |
| Rangjung town and other places | 1 | 9.09 |
| **Have you washed the bite wound with soap and water** |  |  |
| No | 3 | 27.27 |
| Yes | 8 | 72.73 |
| **Have you applied local medicine (seek traditional healer) to the bite wound** |  |  |
| No | 8 | 72.73 |
| Yes | 3 | 27.27 |
| **Have you visited hospital for bite wound treatment** |  |  |
| No (because of provoke bite) | 4 | 36.36 |
| Yes | 7 | 63.64 |
| **Have you received rabies vaccine injection at the hospital** |  |  |
| No | 0 | 0 |
| Yes | 7 | 100 |
| **Whether you asked for the vaccine at the hospital** |  |  |
| I resquested for vaccination | 4 | 57.14 |
| Health staff provided the vaccine | 3 | 42.86 |
| **Have you completed the vaccination course** |  |  |
| No | 0 | 0 |
| Yes | 7 | 100 |
| **In the hospital, whether you received first aid wound management (bite wound washing)** |  |  |
| Yes | 6 | 85.71 |
| Do not remember | 1 | 14.29 |
|  |  |  |
| **Part IV: Attitude and practice of the respondents (missing data= 3)** |  |  |
|  |  |  |
| **Variables** | **N** | **Percent** |
| **What will you do if bitten or scratched by animals** |  |  |
| Wash bite wound with soap and water | 4 | 6.2 |
| Directly visit the hospital | 16 | 25 |
| Both of the above | 40 | 62.5 |
| Both of the above plus contact local healer | 4 | 6.2 |
| **When will you visit hospital after exposure /bitten by animals** |  |  |
| On the same day of bite/exposure | 64 | 100 |
| Next day | 0 | 0 |
| Within 1 week | 0 | 0 |
| Witin 2 week | 0 | 0 |
| Anty time | 0 | 0 |
| Do not know | 0 | 0 |
| **Will you report to the authority in case of any suspected rabies in your community** |  |  |
| No | 1 | 1.56 |
| Not sure | 3 | 4.69 |
| Yes | 60 | 93.75 |
| **Will you kill rabies suspected/rabid dog seen in your community** |  |  |
| No | 30 | 46.88 |
| Not sure | 11 | 17.19 |
| Yes | 23 | 35.94 |
| **Do you advise children not to play with dogs and cats** |  |  |
| No | 4 | 6.25 |
| Yes | 60 | 93.75 |
| **Do you feed stray dogs/free-roaming dogs** |  |  |
| No | 30 | 46.88 |
| Yes | 34 | 53.12 |
| **Is stray dog a problem in the community** |  |  |
| No | 6 | 9.38 |
| Yes | 58 | 90.63 |
| **What do you think should be an approach to control rabies in Rangjung town** |  |  |
| Do nothing | 1 | 1.49 |
| Kill all stray dogs in the town | 11 | 16.42 |
| Catch the dogs and impound in one place | 30 | 44.77 |
| Catch the dogs and relocate to other places | 4 | 5.97 |
| Conduct emergency vaccination | 9 | 13.43 |
| No idea (do not know) | 12 | 17.91 |
|  |  |  |
| **Part V: Dog and cat ownership among the respondents in Rangjung town** |  |  |
|  |  |  |
| **Variables** | **N** | **Percent** |
| **Do you own dog** |  |  |
| No | 60 | 89.55 |
| Yes | 7 | 10.45 |
| **Do you own cat** |  |  |
| No | 61 | 91.04 |
| Yes | 6 | 8.96 |
| **Type and number of dogs** |  |  |
| Local breed male | 2 |  |
| Local breed female | 2 |  |
| Improved breed male | 1 |  |
| Improved breed female | 3 |  |
| **Source of dogs** |  |  |
| Adopted stray dogs | 2 | 28.57 |
| Given by friends | 1 | 14.29 |
| Purchased from others | 4 | 57.14 |
| **Purpose of keeping dogs** |  |  |
| As pet | 4 | 57.14 |
| To guard properties | 3 | 42.86 |
| **Dog management system /ownership** |  |  |
| Restrict movement and keep within home premise | 3 | 42.86 |
| Roam outside day and night | 3 | 42.86 |
| Both of the above | 1 | 14.29 |
| **Is your dog registered with the veterinary centre** |  |  |
| No | 5 | 71.43 |
| Yes | 2 | 28.57 |
| **Have your dog been vaccinated against rabies** |  |  |
| No | 1 | 14.29 |
| Yes | 6 | 85.71 |
| **Have knowedge on the importance of annual vaccination of dogs against rabies** |  |  |
| No | 1 | 14.29 |
| Yes | 6 | 85.71 |
| **Have you taken your dog to other places and brought back to Rangjung recently** |  |  |
| No | 7 | 100 |
| Yes | 0 | 0 |
